# The HIV patient profile in 2013 and 2003: Results from the Greek AMACS cohort

**DOI:** 10.1371/journal.pone.0203601

**Published:** 2018-09-12

**Authors:** Nikos Pantazis, Maria Chini, Anastasia Antoniadou, Helen Sambatakou, Athanasios Skoutelis, Panagiotis Gargalianos, Sophia Kourkounti, Charalambos Gogos, George Chrysos, Mina Psichogiou, Nikolaos V. Sipsas, Olga Katsarou, Periklis Panagopoulos, Simeon Metallidis, Giota Touloumi, on behalf of the AMACS

**Affiliations:** 1 Department of Hygiene, Epidemiology and Medical Statistics, Medical School, National and Kapodistrian University of Athens, Athens, Greece; 2 3^rd^ Department Of Internal Medicine—Infectious Diseases Unit, Red Cross General Hospital, Athens, Greece; 3 4^th^ Department Of Internal Medicine, Attikon University General Hospital, Medical School, National and Kapodistrian University of Athens, Athens, Greece; 4 HIV Unit, 2^nd^ Department of Internal Medicine, Hippokration General Hospital, Medical School, National and Kapodistrian University of Athens, Athens, Greece; 5 5^th^ Department of Internal Medicine—Division of Infectious Diseases, Evangelismos General Hospital of Athens, Athens, Greece; 6 1^st^ Department of Internal Medicine and Infectious Diseases Unit, G. Gennimatas General Hospital, Athens, Greece; 7 AIDS Unit, Clinic of Venereologic & Dermatologic Diseases, Syngros Hospital, Athens, Greece; 8 Department of Internal Medicine & Infectious Diseases, Patras University General Hospital, Patras, Greece; 9 Infectious Diseases Unit, Tzaneion General Hospital of Piraeus, Athens, Greece; 10 1^st^ Department of Propaedeutic Medicine, Medical School, National and Kapodistrian University of Athens, Athens, Greece; 11 Infectious Diseases Unit, Department of Pathophysiology, Laikon Athens General Hospital and Medical School, National and Kapodistrian University of Athens, Athens, Greece; 12 Blood Centre, National Reference Centre for Congenital Bleeding Disorders, Laikon Athens General Hospital and Medical School, National and Kapodistrian University of Athens, Athens, Greece; 13 Infectious Diseases Unit, 2^nd^ University Department of Internal Medicine, University Hospital of Alexandroupolis, Democritus University of Thrace, Alexandroupolis, Greece; 14 1^st^ Internal Medicine Department, Infectious Diseases Unit, Ahepa University Hospital, Aristotle University of Thessaloniki, Thessaloniki, Greece; Azienda Ospedaliera Universitaria di Perugia, ITALY

## Abstract

Combined Antiretroviral therapy (cART) has improved life-expectancy of people living with HIV (PLHIV) but as they age, prevalence of chronic non-AIDS related comorbidities may increase. We study the evolution of HIV-disease markers and comorbidities’ prevalence in PLHIV in Greece. Two cross-sectional analyses (2003 and 2013) on data from the AMACS cohort were performed. Comparisons were based on population average models and were repeated for subjects under follow-up at both 2003 and 2013. 2,403 PLHIV were identified in 2003 and 4,910 in 2013 (1,730 contributing for both cross-sections). Individuals in 2013 were on average older, diagnosed/treated for HIV for longer, more likely to be on cART, virologically suppressed, and with higher CD4 counts. Chronic kidney disease, dyslipidemia and hypertension prevalence increased over time. There was an increase in prescription of lipid-lowering treatment (3.5% in 2003 vs. 7.7% 2013, p<0.001). Among 220 and 879 individuals eligible for Framingham 10-year Event Risk calculation, the proportion of patients in the high-risk group (>20%) increased from 18.2% to 22.2% (p = 0.002). Increase in the prevalence of comorbidities was more pronounced in the subset of patients who were followed in both 2003 and 2013. The increased availability and uptake of cART led to significant improvements in the immuno-virological status of PLHIV in Greece, but they aged alongside an increase in prevalence of non-AIDS related comorbidities. These results highlight the need for appropriate monitoring, optimal cART selection and long-term management and prevention strategies for such comorbidities.

## Introduction

The introduction of combined antiretroviral therapy (cART) has produced significant improvements in the outcomes of people living with HIV (PLHIV), with significant gains in individuals’ survival and reduced HIV related morbidity [1–4]. Mainly due to cART efficacy, the number of PLHIV as well as their age has increased during the last decades [5–7]. Moreover, a substantial proportion of individuals acquire HIV at older ages [8], further contributing to the growing number of PLHIV living into their 50s and 60s [9].

Patients’ aging brings new challenges to the infected individuals themselves, physicians and national health providers. Several studies indicate that the duration of HIV infection is associated with various comorbidities, including cardiovascular diseases (CVD), hypertension, type-II diabetes mellitus, chronic kidney disease (CKD), osteopenia/osteoporosis, and non-AIDS malignancies (e.g. lung, anal, liver cancer, Hodgkin lymphoma). The majority of such comorbidities are more prevalent and tend to appear at an earlier age when compared to the general population [10–16]. Besides the infection itself, cART has been related to increased prevalence of dyslipidemia, diabetes mellitus, and insulin resistance [17, 18]. Other potential contributing factors may be the increased prevalence of known risk factors, immune dysfunction and chronic HIV-related immune activation [19–21].

In light of these findings, it is essential to study the HIV epidemic’s evolution, in terms of both risk factors and subsequent comorbidity changes. The present study aims to assess how the evolution of the HIV epidemic during a decade impacts the prevalence of comorbidities and risk factors for those comorbidities by comparing results from two cross-sectional analyses of patients enrolled and followed-up in Athens Multicenter AIDS Cohort Study (AMACS) in 2003 and 2013. The study also focuses on the aging cohort of individuals who were alive and under follow-up in both 2003 and 2013. By analysing changes over 10 years in this subgroup, not only the effect of ageing but also the long-term consequences of HIV infection and of treatment can be investigated. Focus is given to the most common comorbidities (such as CKD, CVD, type-II diabetes mellitus, dyslipidemia and hypertension), related risk factors but also demographics, treatment and disease markers (i.e. CD4 cell count and HIV-RNA viral load). The results of the study may prove useful in the description of the current needs in HIV care with the specificities of the national HIV care landscape and those of people aging with the HIV infection.

## Materials and methods

### Data source: The Athens Multicenter AIDS Cohort Study (AMACS)

Data were derived from the AMACS electronic database. AMACS is a collaborative, population-based cohort study initiated in 1996. Currently, 14 out of 16 clinics that follow PLHIV in Greece, participate in the study. All HIV infected patients seen in one of the collaborating clinics who were either diagnosed before and alive on 1/1/1996 or diagnosed after 1/1/1996 are eligible for inclusion in AMACS, provided they have been followed for at least one year, or are still under follow-up. Patients who died within the first year of prospective follow-up are not excluded from the cohort. In accordance with data protection policy, data are provided by the clinics after de-identification. The study has been approved by the Athens University IRB (http://en.uoa.gr/), the HCIDC IRB (http://www.keelpno.gr/en-us/home.aspx) and the National Organization of Medicines (http://www.eof.gr/web/guest/home).

A standardized protocol is used for data collection. A variety of data are recorded at study entry including demographic characteristics, clinical events and deaths, antiretroviral therapy (including reasons for change and adverse events) and laboratory tests. Data are updated yearly and thoroughly checked for errors and inconsistencies. To date (last data merger January, 2015), data on 7575 subjects (demographic characteristics, clinical events and deaths, antiretroviral therapy, laboratory tests and resistance tests) have been recorded and checked for errors.

### Definitions and formulas

The 10-year risk of CVD was estimated using the Framingham Risk Score Calculator for Coronary Heart Disease [22] whereas the estimated glomerular filtration rate (eGFR), used as a renal function index, was evaluated through the CKD-EPI formula [23]. CKD was defined as having eGFR<60 ml/min/1.73m^2^ whereas eGFR values between 60 and 89 were considered as indicative of mildly reduced kidney function. Diabetes was defined, as fasting glucose ≥ 126 mg/dL, non-fasting glucose > 200 mg/dL, or taking antidiabetic drugs or insulin. Dyslipidemia was defined as elevated total cholesterol ≥240 mg/dl, and/or decreased HDL-cholesterol ≤35mg/dl, and/or elevated triglycerides ≥150 mg/dl, and/or increased LDL-cholesterol≥150 mg/dl, and or total cholesterol/HDL ratio>4, and/or use of a lipid-lowering medication. Hypertension was defined as systolic blood pressure ≥140 mmHg and/or diastolic blood pressure ≥90 mmHg or taking antihypertensive drugs.

### Statistical analysis

Two cross sectional descriptive analyses were performed at two points in time: 2003 and 2013. Individuals, aged ≥18 years at HIV diagnosis, alive and under active follow-up (having at least one clinical visit or treatment initiation/change or any laboratory test/measurement) during 2003 and/or 2013 were eligible for this study. Distributions of categorical variables were summarized through absolute and relative (%) frequencies or graphically through bar plots. For continuous variables, medians and interquartile ranges (IQR) were used. Box-plots were used to graphically summarize the distributions of continuous variables. Comparisons between 2003 and 2013 were performed mainly through population average (marginal) models for repeated measurements, fitted through generalized estimating equations (GEE), in order to take into account the potential correlations between measurements on the same individuals taken in both 2003 and 2013. For unordered categorical variables with more than two levels, ordered categorical variables and non-normally distributed continuous variables multinomial logistic, ordinal logistic and median regression models (with adjustments for repeated measurements on the same individual) were used, respectively. All analyses were repeated on the subset of individuals contributing data to both 2003 and 2013 cross-sections. We use the terms a) “open cohort” and b) “closed cohort” to refer to a) the full sample of all eligible patients contributing data to either one or both cross sections and b) the subset of patients who contributed data to both cross-sections, respectively. Analyses of the “open cohort” data were repeated after excluding individuals infected through intravenous drug use. P-values lower than 0.05 were considered statistically significant. All analyses were performed using Stata 14.2 (Stata Corp., TX USA).

## Results

**Fig 1:**
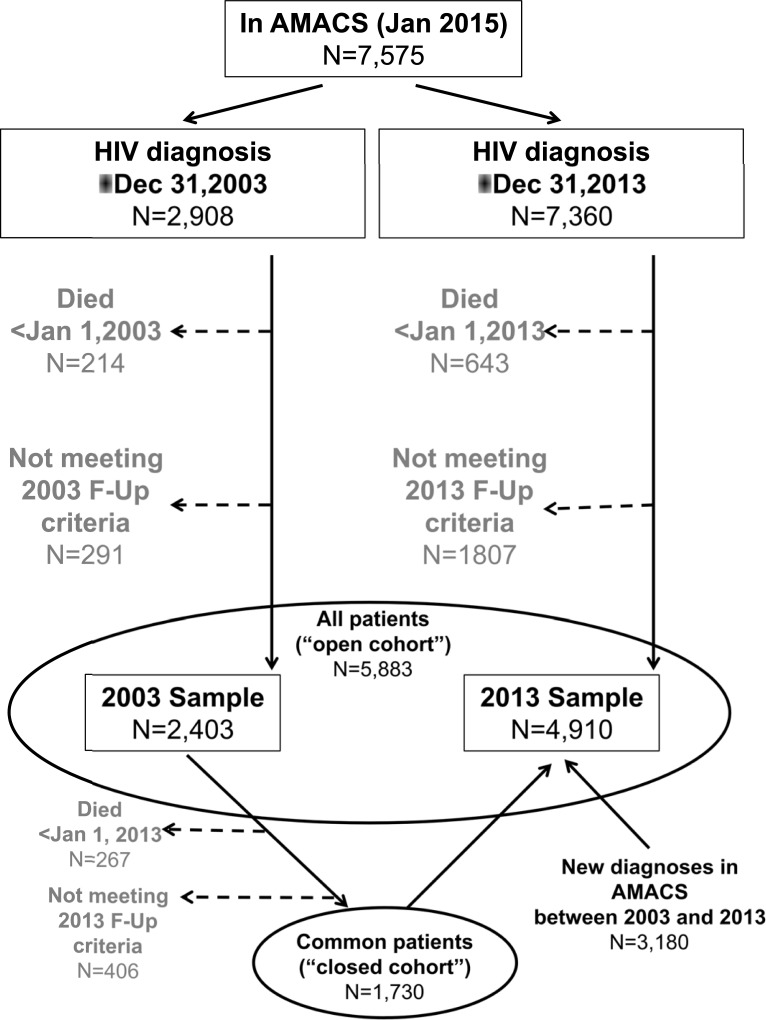
Flow chart for the selection of the 2003 and 2013 cross-sectional samples.

Out of 2,908 AMACS participants who were diagnosed in or before 2003, 2,403 (82.6%) were eligible for inclusion in the 2003 cross-section. The respective number for pre-2013 diagnoses was 7,360, of whom 4,910 (66.7%) were included in the 2013 cross-section. The two cross-sections included in total 5,583 individuals (open cohort) of whom 1,730 (31.0%) contributed data for both years (closed cohort). Within this 10-year period there were 3,180 new patients entering the cohort, whereas 673 of those included in the 2003 sample were not included in the 2013 cross-section (267 died before 2013 and 406 were lost to follow up). Compared to those who remained on follow-up, patients who were lost to follow-up were less likely to be Caucasians, slightly younger and were treated for shorter periods whereas those who died were significantly older, more likely to be heterosexually infected or IDUs, with higher prevalence of AIDS or comorbidities. The most common causes of death were related to cardiovascular diseases (26%), AIDS related conditions (15.7%), non-AIDS malignancies (12.8%) and other non-AIDS related infections (9.8%). Both groups had worse CD4 and viral load levels.The selection process for the two cross-sectional samples is presented graphically in Fig 1.

### Open cohort characteristics and outcomes

Demographic characteristics, disease markers and outcomes of the open cohort are shown in Table 1A. In 2013, patients were older by approximately 3 years on average, compared to those in the 2003 sample. The proportion of those aged over 50 years increased from 18.2% to 26.9% (p<0.001). The proportion of men was slightly higher in 2013, 85.4% *vs*. 82.1% (p<0.001). The increase in the proportion of those infected through intravenous drug use (IVDU) was substantial (from 2.7% to 10.1%; p<0.001). Individuals in the 2013 sample were diagnosed and treated for HIV for a longer duration compared to those in 2003 (6.7 *vs*. 6.0 and 4.5 *vs*. 3.8 years on median, respectively; p<0.001 for both), but the proportion of those who had already progressed to clinical AIDS was lower (13.0% *vs*. 16.2% in 2003; p<0.001). PLHIV were more likely to be on ART (84.4% *vs*. 76.5% in 2003; p<0.001) and typical triple therapy (i.e. two nucleoside reverse transcriptase inhibitors plus a third agent) was more common in 2013 (76.9% *vs*. 63.5% in 2003; p<0.001).

**Table 1:** Demographics, HIV markers and outcomes in 2003 and 2013.

	a) Open cohort					b) Closed cohort				
	2003	NA	2013	NA	p-value	2003	NA	2013	NA	p-value
	(n = 2,403)	(%)	(n = 4,910)	(%)		(n = 1,730)	(%)	(n = 1,730)	(%)	
Median age (years, IQR)	39.4 (33.8, 46.5)	0.0	42.8 (35.2, 50.7)	0.0	<0.001	39.1 (33.8, 45.3)	0.0	49.0 (43.8, 55.3)	0.0	<0.001
Age ≥50	18.2%	0.0	26.9%	0.0	<0.001	15.5%	0.0	46.0%	0.0	<0.001
Gender, male	82.1%	0.0	85.4%	0.0	<0.001	81.8%	0.0	81.8%	0.0	-
Caucasians	95.4%	5.8	96.0%	4.1	0.019	96.5%	4.8	96.5%	4.8	-
Risk Group		14.9		11.9	<0.001		14.7		14.7	
*MSM*	58.4%		62.9%			60.8%		60.8%		-
*IVDU*	2.7%		10.1%			1.8%		1.8%		-
*MSW*	36.3%		26.1%			35.3%		35.3%		-
AIDS diagnosis	16.2%	0.0	13.0%	0.0	<0.001	15.1%	0.0	19.5%	0.0	<0.001
Median time since diagnosis, years (IQR)	6.0 (2.9–9.0)	0.0	6.7 (2.8–13.1)	0.0	<0.001	5.8 (2.8–8.9)	0.0	15.7 (12.7–18.8)	0.0	<0.001
Patients on ART	76.5%	0.0	84.4%	0.0	<0.001	79.1%	0.0	96.1%	0.0	<0.001
Median time on ART, (years, IQR)	3.8 (0.5–6.4)	0.0	4.5 (1.1–11.0)	0.0	<0.001	3.9 0.8–6.5)	0.0	13.6 (10.3–16.4)	0.0	<0.001
Triple therapy (2 NRTI+3rd agent)	63.5%	0.0	76.9%	0.0	<0.001	66.2%	0.0	78.7%	0.0	<0.001
Patients with CD4 count >500 cells/μL	49.5%	17.5	65.7%	5.8	<0.001	54.1%	14.0	71.8%	5.9	<0.001
Patients with viral load <50 copies/mL	41.9%	18.4	75.0%	3.5	<0.001	44.1%	15.0	86.6%	4.2	<0.001
Current smoker	61.0%	70.5	63.4%	58.2	0.106	59.6%	67.6	64.4%	57.6	0.137
Median Framingham risk (%) score (IQR)	9.6 (4.3–17.0)	90.8	8.2 (3.9–18.1)	82.1	0.101	9.6 (3.8–16.9)	89.2	15.2 (8.0–26.6)	81.1	<0.001
High (≥20%) Framingham risk score	18.2%	90.8	22.2%	82.1	0.002	17.2%	89.2	38.2%	81.1	<0.001
Overall cardiovascular events (ever)	1.7%	0.0	2.1%,	0.0	<0.001	1.6%	0.0	3.7%	0.0	<0.001
*Myocardial infarction (ever)*	1.3%	0.0	1.7%	0.0	0.001	1.3%	0.0	3.1%	0.0	<0.001
*Stroke (ever)*	0.3%	0.0	0.4%	0.0	0.102	0.3%	0.0	0.6%	0.0	0.017
Patients with dyslipidemia	64.9%	24.4	70.3%	10.8	<0.001	66.1%	20.1	78.3%	10.7	<0.001
Patients on lipid-lowering treatment	3.5%	0.0	7.7%	0.0	<0.001	4.3%	0.0	14.0%	0.0	<0.001
Patients with hypertension	30.6%	82.7	34.4%	72.3	<0.001	29.2%	81.6	47.3%	72.9	<0.001
Patients on antihypertensive treatment	2.2%	0.0	3.0%	0.0	<0.001	2.0%	0.0	5.4%	0.0	<0.001
eGFR <60 ml/min/1.73m^2^	2.4%	25.3	3.4%	11.9	0.006	1.1%	21.0	4.9%	12.9	<0.001
eGFR 60–89 ml/min/1.73m^2^	25.9%	25.3	24.6	11.9	0.753	24.0%	21.0	29.4	12.9	<0.001

Percentages calculated after exclusion of missing values; IQR: interquartile range; AIDS: Acquired Immunodeficiency Syndrome; NRTI: nucleotide reverse transcriptase inhibitor; ART: antiretroviral therapy; eGFR: estimated glomerular filtration rate; SD: standard deviation; MSM: men who have sex with men; IVDU: intravenous drug users; MSW: sex between men and women; NA(%) percentage of cases with not available data

Results shown for a) open cohort (all eligible patients) and b) closed cohort (subgroup of common patients in 2003 and 2013).

**Fig 2:**
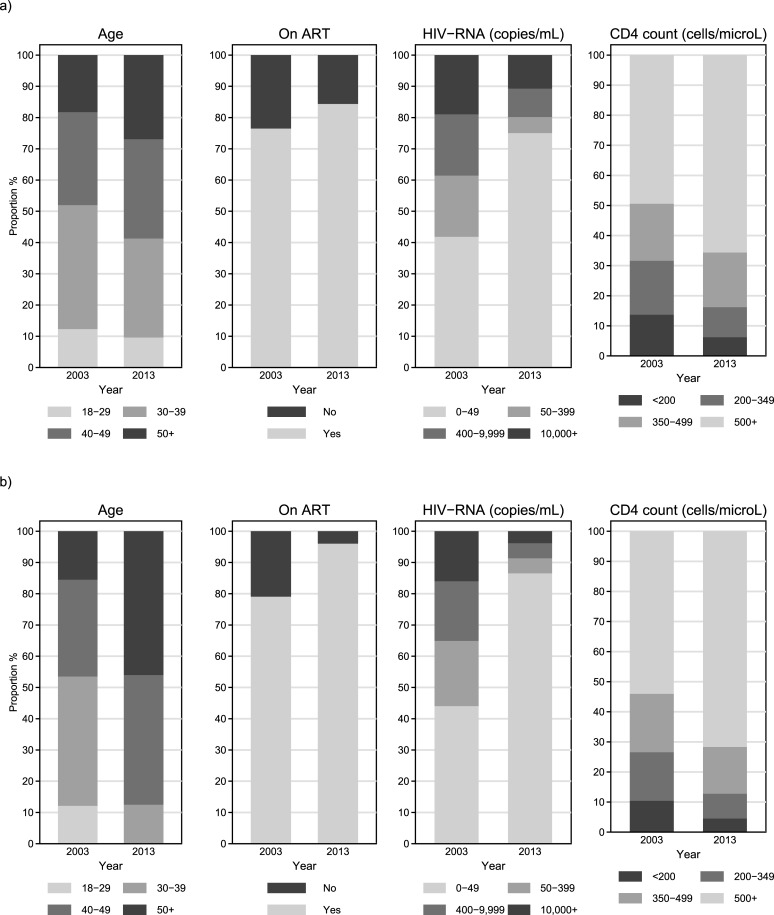
Distribution of age, antiretroviral treatment, HIV-RNA viral load and CD4 cell count in a) open cohort (all eligible patients) and b) closed cohort (subgroup of common patients in 2003 and 2013).

Virologic and immunologic markers were significantly improved in the 2013 sample compared to 2003, with 65.7% of the study participants having >500 CD4 cells/μL (compared to 49.5% in 2003; p<0.001) and 75.0% having undetectable HIV-RNA viral load (compared to 41.9% in 2003; p<0.001). Thirty one patients died during 2003 and 34 during 2013 with the corresponding rates per 1000 person-years being 13.7 (95% CI: 9.6–19.5) and 8.0 (95% CI: 5.7–11.1), respectively (p = 0.059). The distributions of age, antiretroviral treatment, HIV-RNA viral load and CD4 cell count of the overall population in the two time points are graphically summarized in Fig 2A.

**Fig 3:**
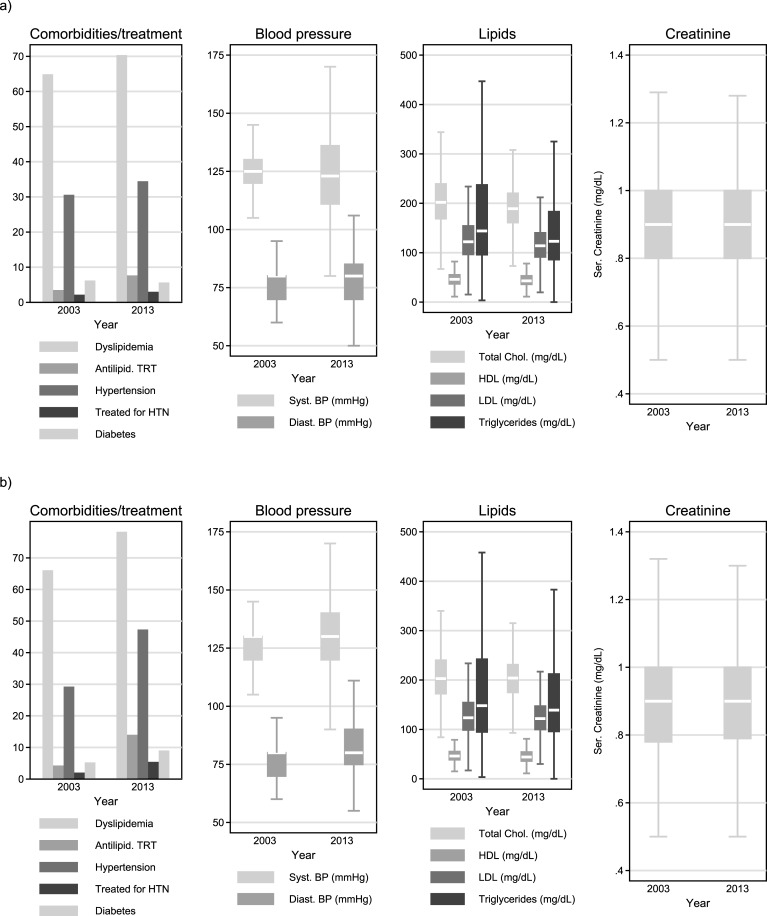
Prevalence of comorbidities, treatment (TRT) for hypertension (HTN) or lipid lowering, blood pressure, lipids and serum creatinine levels in a) open cohort (all eligible patients) and b) closed cohort (subgroup of common patients in 2003 and 2013).

Prevalence of smoking was slightly higher in 2013 (63.4%) compared to 2003 (61.0%) but the difference was not statistically significant (p = 0.101). Systolic and diastolic pressure levels were on average similar in 2013 and 2003 and the proportion of individuals receiving anti-hypertensive treatment increased from 2.2% in 2003 to 3.0% in 2013 (p<0.001) thus the proportion of individuals classified as hypertensive increased significantly (p<0.001) from 30.6% in 2003 to 34.4% in 2013 (Fig 3A). Lipids levels showed modest but statistically significant (p<0.001) decreases in 2013 compared to 2003 but this was probably due to the increase in the percentage of individuals receiving lipid lowering treatment (from 3.5% in 2003 to 7.7% in 2013; p<0.001) thus the already high prevalence of dyslipidemia in 2003 (64.9%) further increased to 70.3% in 2013 (p<0.001) whereas the prevalence of type-II diabetes mellitus declined from 6.2% in 2003 to 5.6% in 2013 (Fig 2A). The proportion of individuals with a previous cardiovascular event (myocardial infarction or stroke) was 1.7% in the 2003 sample and increased to 2.1% 2013 (p<0.001; Table 1A).

The combined effect of these changes on the risk of CVD resulted in a non-statistically significant reduction in the median Framingham risk score (FRS) from 9.6% in 2003 to 8.2% in 2013 (p = 0.101) but with a simultaneous increase in the upper quartile (IQR 4.3%-17.0% in 2003 *vs*. 3.9%-18.1% in 2013) which resulted in an increased proportion of individuals in the high-risk (FRS≥20%) category (from 18.2% in 2003 to 22.2% in 2013; p = 0.002; Table 1A and Fig 3A). It should be noted though, that data for FRS calculation in 2003 and 2013 were available for 220 and 879 individuals, respectively.

**Fig 4:**
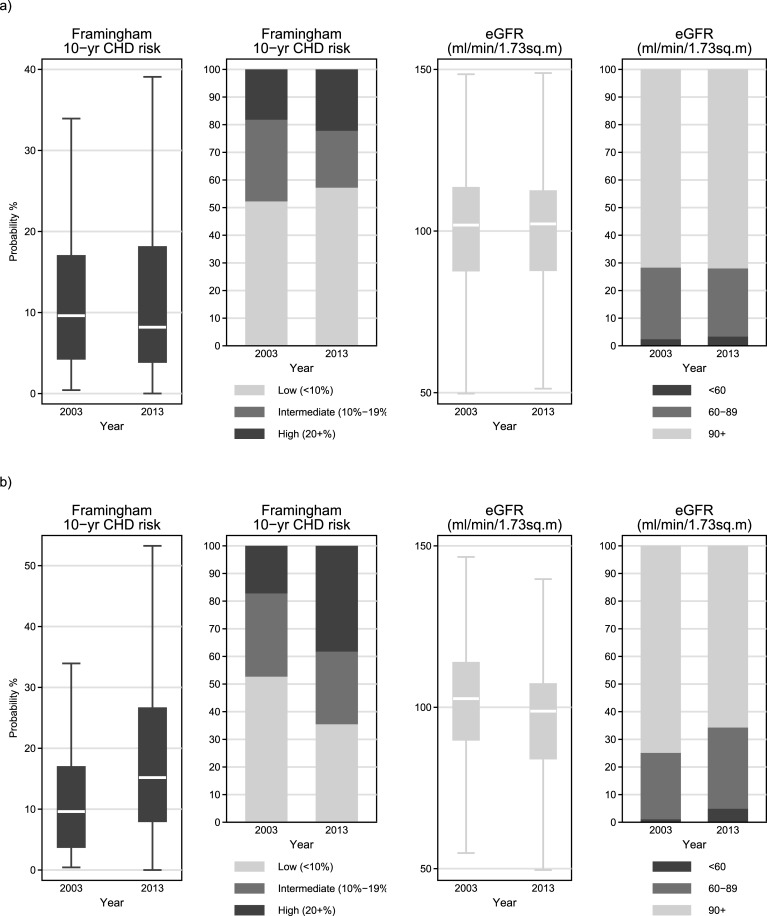
Framingham risk score and estimated glomerular filtration rate (eGFR) quantitatively in categories in a) open cohort (all eligible patients) and b) closed cohort (subgroup of common patients in 2003 and 2013).

Serum creatinine levels seemed stable [Median-IQR 0.9 (0.8, 1.0) mg/dL in both years; Fig 2A], eGFR were only slightly higher in 2013 [Median-IQR 102.2 (87.9, 112.3) *vs*. 101.9 (87.8, 113.4) ml/min/1.73m^2^ in 2013 and 2003, respectively; Fig 3A] but the prevalence of CKD (defined as eGFR<60 ml/min/1.73m^2^) increased significantly (p = 0.006) from 2.4% in 2003 to 3.4% in 2013 (Table 1A and Fig 4A).

**Fig 5:**
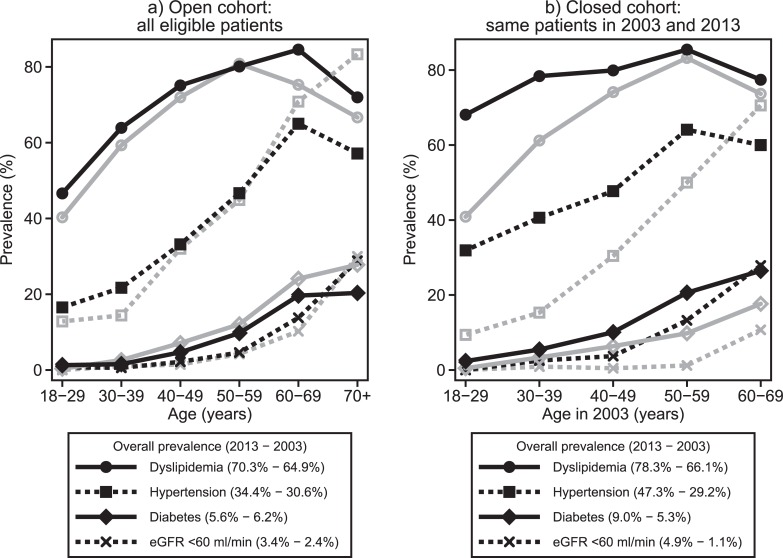
Prevalence of comorbidities in 2013 (black lines) and 2003 (grey lines) by age group in a) open cohort (all eligible patients) and b) closed cohort (subgroup of common patients in 2003 and 2013; results shown by their age in 2003). Six patients, aged 70+ in 2003, not shown in sub-figure b.

Finally, prevalence of all comorbidities was increasing with age and in most cases (with the exception of diabetes), it was slightly higher in all age groups in 2013 compared to the same age groups in 2003 (Fig 5A). Comparing the 2013 sample to the 2003 one, the adjusted for current age Odds Ratios [(95% CIs); p-values] for dyslipidemia, hypertension, diabetes and CKD were 1.20 [(1.07, 1.34); 0.001], 1.29 [(1.01, 1.64); 0.038], 0.75 [(0.62, 0.90); 0.003] and 1.18 [(0.85, 1.65); 0.323], respectively.

Excluding IVDUs from both samples resulted in an increase of 2 years in the median age, slightly higher prevalence of dyslipidemia and hypertension (72.0% and 36.2%, respectively) and better immunologic and virologic outcomes (CD4>500 cells/μL: 68.6%; viral load <50 copies/mL: 77.1%) in the 2013 sample with all the other measured parameters having negligible changes compared to those of the main analysis.

### Closed cohort characteristics and outcomes

Most of the changes observed in the open cohort and described above were of the same direction, yet of increased magnitude in the aging cohort of patients who contributed data for both cross sections (alive and under follow-up in both 2003 and 2013).

As shown in Table 1B, the average age in 2003 was 39.1 years and only 15.5% of patients in the cohort were over 50 years old whereas in 2013 almost half of the cohort (46.0%) was more than 50 years old. An additional 4.4% had progressed to clinical AIDS during this decade (from 15.1% in 2003 to 19.5% in 2013; p<0.001). Almost all of them (96.1%) were on ART in 2013, and despite being diagnosed for a median of almost 16 years, 71.8% had more than 500 CD4 cells/μL and 86.6% had undetectable HIV-RNA viral load (compared to 54.1% and 44.1% in 2003, respectively; Fig 2B; p<0.001). The percentage of smokers increased in this subgroup (from 59.6% in 2003 to 64.4% in 2013) but the difference was not statistically significant (p = 0.137). Systolic and diastolic pressure levels showed statistically significant increases from 2003 to 2013 (Fig 3B) as did the proportions of patients receiving treatment for hypertension (2.0% in 2003 and 5.4% in 2013; p<0.001) or classified as hypertensive (29.2% in 2003 and 47.3% in 2013; p<0.001). Lipids levels (Fig 3B) and their respective changes within the 2003–2013 decade were similar to those observed in the open cohort but the proportion of those on lipid-lowering treatment had increased (from 4.3% in 2003 to 14.0% in 2013; p<0.001) thus 78.3% of the cohort was classified as dyslipidemic in 2013 compared to 66.1% in 2003 (p<0.001). The prevalence of type-II diabetes mellitus in this subgroup showed a significant increase from 5.3% in 2003 to 9.0% in 2013 (p<0.001). The proportion of individuals with a previous cardiovascular event (myocardial infarction or stroke) was 1.6% in the 2003 sample and increased to 3.7% in the 2013 one (p<0.001; Table 1B).

The average FRS of this subgroup was 9.6% (IQR 3.8%-16.9%) in 2003 and it increased significantly (p<0.001) to 15.2% (8.0%-26.6%) in 2013. The proportion of individuals in the high FRS category (FRS≥20%) increased (p<0.001) from 17.2% in 2003 to 38.2% in 2013 (Fig 4B). The FRS calculations were possible for 186 and 327 individuals from this subgroup in 2003 and 2013, respectively.

Serum creatinine levels seemed stable [Median-IQR 0.9 (0.8, 1.0) mg/dL in both years; Fig 3B] but eGFR decreased significantly (p<0.001) within the decade [Median-IQR 102.8 (89.9, 113.8) *vs*. 98.8 (84.1, 107.2) ml/min/1.73m^2^ in 2003 and 2013, respectively; Fig 3B] and the prevalence of CKD (i.e. as eGFR<60 ml/min/1.73m^2^) increased also significantly (p<0.001) from 1.1% in 2003 to 4.9% in 2013 (Table 1B and Fig 3B). Significant (p<0.001) increases were also observed in the proportion of patients with mildly reduced kidney function (i.e. with eGFR between 60 and 89 ml/min/1.73m^2^).

In Fig 5B, the prevalence of dyslipidemia, hypertension, diabetes and CKD is shown according to the patients’ age in 2003. Increases were observed in all comorbidities and age groups. Dyslipidemia and hypertension prevalence increases were more intense among patients who were relatively younger whereas increases in the prevalence of diabetes and CKD were more noticeable among older patients. For example comparing 2013 to 2003 and restricting the sample to those aged less than 40 years in 2003, the Odds Ratios [(95% CIs); p-values] for dyslipidemia and hypertension were 2.38 [(1.97, 2.86); <0.001] and 4.53 [(2.48, 8.27); <0.001], respectively. The corresponding Odds Ratios [(95% CIs); p-values] for diabetes and CKD, for patients aged 40 years or more in 2003, were 1.84 [(1.47, 2.30); <0.001] and 5.45 [(3.00, 9.91); <0.001], respectively.

## Discussion

In this study we used data from a large multicenter observational database in order to investigate changes, within the decade 2003 to 2013, in the profile of PLHIV who were diagnosed and linked to care in Greece.

Our results confirmed the increase in the average age of PLHIV seen in clinics as approximately one over four out of the 2013 sample was more than 50 years old and the proportion was one over two in the subset of those who were under follow-up in 2003 and kept on being under follow-up in 2013. Sex between men was the most frequent mode of transmission in both years but the proportion of those infected through intravenous drug use was almost quadrupled, most probably due to the 2011–2012 HIV outbreak in IVDUs in Athens [24]. It is noteworthy though that increased proportion of IVDUs in the 2013 sample of the open cohort had very limited effects on the main findings.

The increased use of triple therapy antiretroviral regimens as standard of care, with the introduction of new antiretroviral agents and the increased cART uptake led to significant immuno-virological improvements in the decade between 2003 and 2013 and reduced death rates. Almost two thirds of the open cohort in 2013 had CD4 counts of greater than 500 cells/μL, and 75% had undetectable HIV-RNA viral load. Immuno-virological status was even better in the subgroup of common in 2003 and 2013 patients. Moreover, the proportion of patients who had progressed to AIDS in the open cohort was lower in 2013 compared to 2003. The increase in the corresponding proportions in the closed cohort was unfortunately unavoidable as an additional number of patients (below 5%) who were AIDS free in 2003, progressed to AIDS within the 2003–2013 decade.

The AMACS population aged alongside an increase in prevalence of non-HIV related chronic comorbidities, such as hypertension, dyslipidemia and CKD despite the high proportion of new patients who entered the cohort over time. These increases were particularly evident for the aging cohort of patients who were captured in the data cohorts in both years (2003, 2013), a sample which reflects better the HIV population moving forward as it not impacted by the entry of new patients into the cohort. Thus, in the closed cohort over the 10 year period an increase in all studied comorbidities was observed, with younger age groups (those under 40 in 2003) particularly contributing to the increase in dyslipidemia and hypertension and older age groups to increase in diabetes and CKD. This outcome was observed despite the potentially improved disease prognosis a potential survivor bias might be expected to produce. The open cohort patients managed to keep lipid and blood pressure levels relatively stable through medical management. It should be noted though, that the proportions of patients who were on anti-lipid or anti-hypertensive treatment seem low. A degree of underreporting in non-ART drugs cannot be excluded but given the established benefits of such treatments we expect to observe increased administration in post-2013 data. Smoking prevalence had not decreased, which in combination with the increased prevalence of type-II diabetes mellitus and aging itself led to an increase in the proportion of patients with high (≥20%) 10-year risk for coronary heart disease as estimated by the Framingham risk score. This was notable in the subgroup of the aging subgroup, where this proportion reached approximately 40% in 2013. Additionally, a non-negligible proportion of the study participants, especially among those in the closed cohort, had already experienced a myocardial infarction or a stroke. It is worth mentioning that the corresponding data reported in 2013 are cumulated with the events recorded in 2003 thus the differences in the closed cohort reflect events which occurred between 2003 and 2013. Finally, we also found increases in the proportions of those with at least mild to moderate loss of kidney function indicating a possible increase in the prevalence of chronic kidney disease. However, the drop in median eGFR levels observed in the closed cohort is not far from what would be expected due to the aging of the participants alone[25].

These findings are in general consistent with those reported by other researchers in similar settings but comparative studies between different time points are rare. A study in Australia, comparing HIV infected men in 1998 and 2010 [26], reported similar trends in FRS and lipid levels. A study based on the 2013 data from the Dutch ATHENA study [27] found slightly lower proportion of subjects in the high (≥20%) FRS category (16% *vs*. 22% in our study) but the prevalence of smoking was much lower compared to our study. A cross-sectional study based on 2011 data from 10 HIV units in Spain [28] found comparable prevalence of smoking and hypertension but lower prevalence of dyslipidemia using though a more strict definition. A USA study of approximately 2,000 PLHIV assessed between 2002 and 2009 estimated that 20% had a high (≥20%) FRS [29], a proportion which is very close to the corresponding estimate in our study. A comparative study of newly enrolled PLHIV in 2005 and 2011 in Italy found similar trends in FRS and lipids levels as those observed in our study [30] and another Italian study estimated a prevalence of type-II diabetes mellitus which was close to our estimates [31]. Finally, increasing rates of CKD have been reported in both Europe [32] and the USA [33]. On the other hand, the large multicenter international START trial [34] reported lower FRS scores (median 4%) and prevalence of hypertension (19.3%) or dyslipidemia (8.9%) for European participants but participants in the START trial were younger, with much shorter durations of HIV infection compared to the participants of our study and ART-naive at the time point considered in the specific study.

Our study is characterized by a rich data source: AMACS includes approximately 70% of the population of diagnosed PLHIV in Greece and is, to a large degree, representative of that population [35]. The long follow-up of AMACS allowed us to compare the profile of the average HIV patient in two time points ten years apart, but also to assess the changes within an aging subcohort of individuals seen at the collaborating clinics continuously from at least 2003 to 2013. The data we used come directly from the patients’ records at each collaborating HIV clinic or the respective hospital databases and are recorded and merged in a central electronic database.

One of the strong assets our study is the ability to highlight both the current needs of the cohort in total but also the individual needs of those aging with HIV who are additionally affected by longer duration of the infection and prolonged ART exposure.

There are some limitations in our study, stemming mainly from the lack of some additional types of data or the presence of missing values. For example, FRS calculation was only possible for a subset of the study participants due to the lack of blood pressure measurements or HDL-cholesterol measurements especially in the 2003 sample. The use of FRS, instead of other HIV-specific formulas [36] to quantify CVD risk has been a subject of debate but some recent studies argue that the Framingham equations may still be preferable [37, 38]. Bone mass density measurements were very rare in practice; thus, we were not able to assess rates of osteopenia/osteoporosis and their respective changes over time. Due to the cross-sectional nature of the study, distinction between the effects of aging and duration of the infection was also not possible. However, we also analysed the changes over a 10 years period in patients who were in care during 2003 and 2013. Interestingly, not only the major findings from the open (cross-sectional) cohort were confirmed in this subgroup, but also diabetes prevalence and Framingham risk score increased significantly, reflecting not only the effect of aging, but also the long-term consequences of HIV infection and treatment. The improvement in the efficacy and tolerability of antiretroviral treatment has clearly changed the prognosis of PLHIV thus the focus of HIV clinicians has partly shifted towards other, non-AIDS but probably HIV related conditions. Although continuous efforts are being made in AMACS to collect complete and reliable data, this shift may have some effect on our results as it is probably associated with a higher attention to test for and record such conditions during the more recent years.

Our results highlight the impressive immunological and virological improvements induced by the modern antiretroviral treatment but at the same time underline the increased frequency of serious and potentially life threatening comorbidities and risks. As some of the comorbidities observed in our cohort may be associated with treatment, cART choices become very important as PLHIV live longer, and thus accumulate greater long-term exposure.

The shift in HIV epidemic paradigm should be addressed with appropriate monitoring and holistic management of HIV care, in terms of optimal cART selection, and long-term management and prevention of comorbidities potentially leading to a continuous improvement of the overall health status of PLHIV. In this context, effective cART options that balance HIV outcomes with less long-term impact on cardiovascular and renal diseases would be beneficial to patient’s care and treatment.
